# Subgingival microbiota in a population with and without cognitive dysfunction

**DOI:** 10.1080/20002297.2020.1854552

**Published:** 2021-01-15

**Authors:** Jacob Holmer, Velma Aho, Maria Eriksdotter, Lars Paulin, Milla Pietiäinen, Petri Auvinen, Marianne Schultzberg, Pirkko J. Pussinen, Kåre Buhlin

**Affiliations:** a Unit of Periodontology, Department of Dental Medicine, Karolinska Institutet, Huddinge, Sweden; b DNA Sequencing and Genomics Laboratory, Institute of Biotechnology, University of Helsinki, Helsinki, Finland; c Department of Neurology, Helsinki University Hospital, Helsinki, Finland; d Division of Clinical Geriatrics, Department of Neurobiology, Care Sciences and Society, Karolinska Institutet, Huddinge, Sweden; e Theme Aging, Karolinska University Hospital, Huddinge, Sweden; f Oral and Maxillofacial Diseases, University of Helsinki and Helsinki University Hospital, Helsinki, Finland; g Division of Neurogeriatrics, Department of Neurobiology, Care Sciences and Society, Karolinska Institutet, Stockholm, Sweden

**Keywords:** Dementia, molecular epidemiology, oral microbiome, periodontal disease, periodontal-systemic disease interactions, RNA sequencing

## Abstract

**Aim**: The aim of this study was to compare the subgingival microbiota of people with Alzheimer´s disease (AD), mild cognitive impairment (MCI), subjective cognitive decline (SCD) and cognitively healthy individuals.

**Materials and methods**: The study population was recruited from 2013 to 2017 and comprised 132 cases recently diagnosed with AD (n = 46), MCI (n = 40) or SCD (n = 46), and 63 cognitively healthy controls. Subgingival samples were collected, and the microbiotas were characterized by *16S rRNA* gene sequencing.

**Results**: The relative abundance of the ten most common genera did not differ between the cases and control groups. However, the microbial richness and evenness were higher in cases than in controls and differed across the four groups. The variables with the greatest influence on the microbial community composition were related to periodontal disease followed by body mass index, study group affiliation and smoking. Ten taxa exhibited significant differences between case participants and controls. Two Operational Taxonomic Units were particularly abundant in AD compared to controls: *Slackia exigua*, which was also associated with deep periodontal pockets, and a *Lachnospiraceae* [G-7] bacterium.

**Conclusion**: It is concluded that in individuals with cognitive impairment or AD, the subgingival microbiota exhibits shifts typical of periodontal disease.

## Introduction

Alzheimer´s disease (AD) is the most prevalent of the dementia disorders and leads to multidomain cognitive dysfunction [1]. The mechanisms underlying AD pathophysiology, leading to brain atrophy and impaired cognitive functions, are not fully understood [2]. Despite considerable progress in identifying dementia risk factors in recent years [3], much of the risk remains unexplained. Preventive healthcare may lead to a reduction in dementia incidence [4]. Thus, the identification of modifiable risk factors and preventive factors relevant to dementia can have important public health implications.

Whether infectious agents represent a true causal effect in AD aetiology has been debated for several decades [5]. Recently, it has been proposed that perturbations or alterations of the intestinal microbiome play a role in AD development [6]. Changes in the gut microbial community may alter the permeability of the gut barrier and induce systemic inflammation through several different pathways [7].

Periodontitis, a complex chronic oral inflammatory disease, has been associated with dementia in observational and experimental studies [8]. The distinctive pathological features of periodontitis are inflammation of the periodontal tissues and subgingival microbiota dysbiosis [9,10]. Both components drive disease progression, eventually leading to loss of tooth-supporting tissues and ultimately to loss of teeth [11]. It has been demonstrated that transmission and colonisation of oral bacteria can be extensive and can influence the gut microbiota [12]. Indeed, the oral cavity is the starting point of the gastrointestinal tract. Moreover, in a number of studies *Porphyromonas gingivalis*, a bacterium associated with periodontitis, has been implicated in dementia [13,14]. It has been suggested that *P. gingivalis* is capable of modulating the gut microbiome [15].

The aim of the present study was to compare the subgingival microbiota in individuals with cognitive dysfunction and in cognitively healthy individuals. We hypothesized that differences in the subgingival microbiota exist between individuals with and without cognitive dysfunction.

## Materials and methods

### Study design

This explorative study of the human oral subgingival microbiota was based on biological samples and data collected as part of a recent case-control study, conducted in Sweden from 2013 to 2017. The study design and preliminary findings have been published previously [16]. Critical components of relevance relating to this investigation are outlined in the following section. The study is reported in compliance with the STROME-ID Statement guidelines [17]. Ethical approval was obtained from the Regional Ethical Review Board in Stockholm (2012/652–31/1). The study conforms to institutional and international (Declaration of Helsinki) standards. Written informed consent was obtained from all participants.

### Data collection

The study population comprised 154 case participants (50–80 years old) diagnosed with AD (n = 52), mild cognitive impairment (MCI [n = 51]) or subjective cognitive decline (SCD [n = 51]); 76 individuals served as cognitively healthy controls. Case participants were enrolled from the Karolinska Memory Clinic at the Karolinska University Hospital in Huddinge, Sweden, after diagnosis at a multidisciplinary consensus conference, using diagnostic data from an extensive medical and neurocognitive assessment. Diagnostic criteria for AD were mainly based on the NIA-AA diagnostic guidelines due to probable AD [18]. MCI diagnosis was set in accordance with the Winblad criteria [19] and for SCD the pre-MCI SCD criteria were used [20].

Controls were frequency-matched for age and sex, identified and enrolled from the population register in Huddinge, Sweden. To be considered eligible, the controls were required not to have sought medical attention for memory loss or experienced memory loss. In addition, controls underwent cognitive screening (excluded if < 28 on the Mini-Mental State Examination [MMSE] test and/or if failing the clock-drawing test [CDT]) before inclusion.

The following exclusion criteria were applied to all participants, *i.e*. case participants and controls alike: severe medical conditions *i.e*. brain tumours, clinically significant liver, kidney or lung dysfunction, chronic inflammatory disease and psychiatric diseases [16]. All study participants completed a questionnaire about personal data (medical, dental, financial, educational information *etc*.).

Following case inclusion and control recruitment, all participants underwent a dental examination at the Department of Dental Medicine, Karolinska Institutet, Huddinge, Sweden. The dental examination comprised a comprehensive assessment of the oral soft and hard tissues including a periodontal examination and a panoramic radiograph using a ProMax® (Planmeca Oy, Helsinki, Finland) according to a standard protocol. Probing pocket depth (PPD) was measured in millimeters using a periodontal probe (UNC 15, Hu-Friedy, Chicago, IL) at six sites on all existing teeth. Bleeding on pocket probing (BoP) was also recorded at six sites on all existing teeth. Radiographically verified marginal alveolar bone loss was classified in the following manner: no/mild (loss of supporting bone < 1/3 of the root length), localized (loss of supporting bone tissue ≥ 1/3 the root length in < 30% of the teeth) or generalized (loss of supporting bone tissue ≥ 1/3 the root length in ≥30% of the teeth). The variable PPD ≥ 6 mm was defined as having ≥1 site with PPD of ≥6 mm.

After completion of the periodontal examination, the deepest or the most representative periodontal pocket was selected in each quadrant for subgingival microbial sampling. One sample was collected per quadrant. If no teeth or dental implants were present in the quadrant, no sample was collected. The quadrant to be sampled was isolated with cotton rolls/pads and a saliva ejector. Supragingival plaque was carefully removed at the selected sampling site using a sterile curette, leaving the subgingival dental biofilm undisturbed. All samples were taken with a new individual sterile curette with a single pull, in a coronal direction, from the base of the periodontal pocket. The procedure was repeated in all four quadrants. The samples were pooled in 1.5 mL microcentrifuge tubes (Thermo Fisher Scientific®) containing PCR grade water (Roche®) and stored at −80°C until further processing.

### Sample processing and microbiome profiling

DNA extraction, PCR amplification and sequencing of the V3-V4 regions of the *16S rRNA* gene were undertaken at the DNA Sequencing and Genomics Laboratory of the Institute of Biotechnology, University of Helsinki, following a previously published protocol [21] and primers [22]. Dual-indexes, selected with BARCOSEL [23], were added to samples in the second PCR step. All samples were combined in a single pool and sequenced twice with Illumina MiSeq (paired-end; read lengths: forward: 326; reverse: 278).

The raw data consisted of 35, 171, 514 sequence reads, which are available in the European Nucleotide Archive (accession: PRJEB35923). Primers were trimmed from reads with cutadapt (v. 1.8.3 [24]). Further quality control, taxonomic classification and Operational Taxonomic Unit (OTU; a computational equivalent to species) clustering were undertaken with mothur [25] following the standard operating procedure (SOP) for MiSeq data [26,27]^40^. The reference databases were Silva (v. 132) for alignment and HOMD (v. 15.1 [28]) for taxonomy. Singleton sequences were removed from data during the mothur analysis.

### Statistical data analysis

Statistical analysis of clinical data was undertaken in Stata (StataCorp. 2019. Stata Statistical Software: Release 16. College Station, TX: StataCorp LLC). The limit for statistical significance was set at 5%. Contingency tables were created to explore frequency distribution of case participants and controls in categories of demographic, socioeconomic and dental variables. Intergroup comparisons were determined by a chi-squared test or Fisher’s exact test for categorical variables and Mann-Whitney test or Kruskall-Wallis test for continous variables.

Statistical analyses involving microbial data were performed in R (v. 3.6.2; R Core Team, 2019). OTUs with fewer than six sequence reads in the entire data were removed. The R package decontam (v. 1.6.0 [29]) was used for detecting contaminant taxa, making use of negative control samples; the contaminants and control samples were trimmed out. Samples with a low number of sequence reads (<24,000), or an unusually high number of sequence reads (>120,000) were also trimmed out. After these steps, the final number of good quality sequences was 16, 048, 390 (mean ± SD: 77, 528 ± 20, 889 per sample).

Phyloseq (v. 1.30.0 [30]) was used for data management and calculating alpha diversity measures (observed richness and Shannon index). Alpha diversity comparisons included Kruskal-Wallis tests (initial testing for categorical variables), Pearson correlations (initial testing for continuous variables), Wilcoxon rank-sum tests (post hoc testing between diagnostic groups) and linear regression (combined model with multiple variables). The R package vegan (v. 2.5–6 [31]) was used for beta diversity analysis: contrasting Bray-Curtis dissimilarities with PERMANOVA and visualization with non-metric multidimensional scaling (NMDS). All diversity comparisons were run on microbiota data subsampled to the smallest number of reads in a sample (24 275).

Differences in abundance of specific bacterial taxa were tested with DESeq2 ([v. 1.26.0 [32]) focusing on OTUs, genera and families that had more than one read in more than one fourth of the samples. All case participants versus controls (two study groups) and diagnostic case subgroups versus controls (all four study groups) were tested with adjustment for age, body mass index (BMI) and PPD ≥ 6. Current smokers (n = 12) were excluded from the DESeq2 comparisons.

The purpose of the study was not to describe the microbiota in periodontitis. Since PPD ≥ 6 mm was found to be the most influential clinical measure on subgingival microbial diversity, we used this variable as a proxy for periodontal health status.

## Results

### Clinical features of the study population

**Figure 1.**
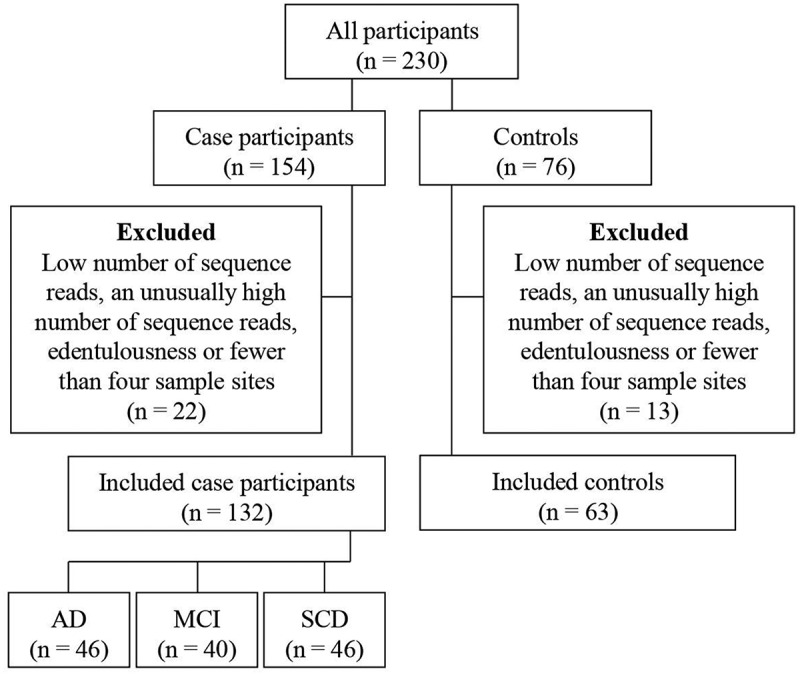
Flow chart demonstrating selection of study participants. **Note**: AD: Alzheimer´s disease. MCI: Mild cognitive impairment. SCD: Subjective cognitive decline

After a careful quality control, the final study sample in this analysis comprised 195 (84.8% of the original population) participants (63 controls and 132 case participants) with complete subgingival microbiome information (Figure 1). The diagnostic case subgroups comprised participants diagnosed with AD (n = 46), MCI (n = 40) or SCD (n = 46). Six participants had both dental implants and teeth sites sampled. The reasons for exclusion were a low number of sequence reads (n = 17), an unusually high number of sequence reads (n = 10), edentulousness (n = 1), or fewer than four sampling sites (n = 7).

Demographic, socioeconomic and clinical data for the participants are presented in (Table 1). The median age of cases was 66 years (interquartile range [IQR] 12) and for controls 69 years (IQR 9). The median age of diagnostic case subgroups was: AD 71 (IQR 11), MCI 70 (IQR 10) and SCD 61 years (IQR 7). Proportion of males was 49.2% among cases and 44.4% among controls and ranged between 45.7% and 52.5% in diagnostic case subgroups. Annual income, marital status, diabetes status, medications, and smoking habits did not differ between cases and controls. However, the control group had higher BMI compared with the cases. The case participants had more often sites with PPD ≥ 6 mm and BoP, and an increased prevalence of marginal alveolar bone loss.

**Table 1.** Distribution of demographic, socioeconomic and clinical characteristics

				Diagnostic case subgroups			
				AD	MCI	SCD	
Variables	Controls n = 63	All cases n = 132	P-value^a^	n = 46	n = 40	n = 46	P-value^b^
	**Median (IQR)**						
**Age (years)**	69 (9)	66 (12)	0.175^c^	71 (11)	70 (10)	61 (7)	0.000^d^
	**n (%)**						
**Sex (male)**	28 (44.4)	65 (49.2)	0.530^e^	23 (50.0)	21 (52.5)	21 (45.7)	0.847^e^
Place of birth (Sweden)*	56 (88.9)	111 (84.7)	0.434^e^	40 (87.0)	35 (89.7)	36 (78.3)	0.358^e^
**Education**							
1–12 years	43 (68.3)	76 (57.6)	0.153^e^	26 (56.5)	31 (77.5)	19 (41.3)	0.003^e^
University	20 (31.8)	56 (42.4)		20 (43.5)	9 (22.5)	27 (58.7)	
**Annual income (SEK/year)**							
< 180 000	11 (17.5)	22 (16.7)	0.133^e^	6 (13.0)	9 (22.5)	7 (15.2)	0.258^e^
180 000–300 000	30 (47.6)	55 (41.7)		24 (52.2)	16 (40.0)	15 (32.6)	
300 001–520 000	11 (17.5)	42 (31.8)		13 (28.3)	12 (30.0)	17 (37.0)	
≥ 520 001	11 (17.5)	13 (9.9)		3 (6.5)	3 (7.5)	7 (15.2)	
**Marital status**							
Married/living together	45 (71.4)	93 (70.5)	0.889^e^	34 (73.9)	28 (70.0)	31 (67.4)	0.919^e^
Single/divorced/widowed	18 (28.6)	39 (29.6)		12 (26.1)	12 (30.0)	15 (32.6)	
**MMSE score**							
0–27	0 (0.0)	52 (39.4)	0.000^e^	34 (73.9)	15 (37.5)	3 (6.5)	0.000^e^
28–30	63 (100.0)	80 (60.6)		12 (26.1)	25 (62.5)	43 (93.5)	
**Diabetes**							
Yes	4 (6.4)	16 (12.1)	0.214^e^	6 (13.0)	4 (10.0)	6 (13.0)	0.560^f^
No	59 (93.7)	116 (87.9)		40 (87.0)	36 (90.0)	40 (87.0)	
**Number of medications**							
0	20 (31.8)	33 (25.0)	0.520^e^	4 (8.7)	13 (32.5)	16 (34.8)	0.083^e^
1–3	27 (42.9)	57 (43.2)		23 (50.0)	16 (40.0)	18 (39.1)	
≥ 4	16 (25.4)	42 (31.8)		19 (41.3)	11 (27.5)	12 (26.1)	
**Body mass index (kg/m^2^)**							
< 18.5	0 (0.0)	1 (0.8)	0.008^f^	1 (2.2)	0 (0.0)	0 (0.0)	0.003^f^
18.5–24.9	22 (34.9)	74 (56.1)		32 (69.6)	20 (50.0)	22 (47.8)	
≥ 25.0	41 (65.1)	57 (43.2)		13 (28.3)	20 (50.0)	24 (52.2)	
**Smoking habits**							
Current	5 (7.9)	7 (5.3)	0.704^e^	4 (8.7)	1 (2.5)	2 (4.4)	0.713^f^
Previous	28 (44.4)	56 (42.4)		22 (47.8)	17 (42.5)	17 (37.0)	
Never	30 (47.6)	69 (52.3)		20 (43.5)	22 (55.0)	27 (58.7)	
**Number of teeth**							
20–32	61 (96.8)	122 (92.4)	0.344^f^	41 (89.1)	37 (92.5)	44 (95.7)	0.360^f^
0–19	2 (3.2)	10 (7.6)		5 (10.9)	3 (7.5)	2 (4.4)	
**Number of sites with PPD ≥6 mm**							
0	52 (82.5)	58 (43.9)	0.000^e^	13 (28.3)	22 (55.0)	23 (50.0)	0.000^e^
≥ 1	11 (17.5)	74 (56.1)		33 (71.7)	18 (45.0)	23 (50.0)	
**Bleeding on pocket probing (% of sites)**							
0–24	49 (77.8)	68 (51.5)	0.002^e^	28 (60.9)	15 (37.5)	25 (54.4)	0.004^f^
25–49	12 (19.1)	53 (40.2)		15 (32.6)	21 (52.5)	17 (37.0)	
50–100	2 (3.2)	11 (8.3)		3 (6.5)	4 (10.0)	4 (8.7)	
**Marginal alveolar bone loss**							
No or mild	42 (66.7)	75 (56.8)	0.113^e^	23 (50.0)	23 (57.5)	29 (63.0)	0.239^f^
Localized	20 (31.8)	45 (34.1)		17 (37.0)	15 (37.5)	13 (28.3)	
Generalized	1 (1.6)	12 (9.1)		6 (13.0)	2 (5.0)	4 (8.7)	

^a^Two-group comparison (combined case participants group and control group) ^b^Four-group comparison (case subgroups and control group) ^c^Mann-Whitney test ^d^Kruskal-Wallis test ^e^Chi-squared test of homogeneity ^f^Fisher’s exact test * Based on 194 individuals. There was one missing value in the MCI group.

### Microbial diversity

**Figure 2.**
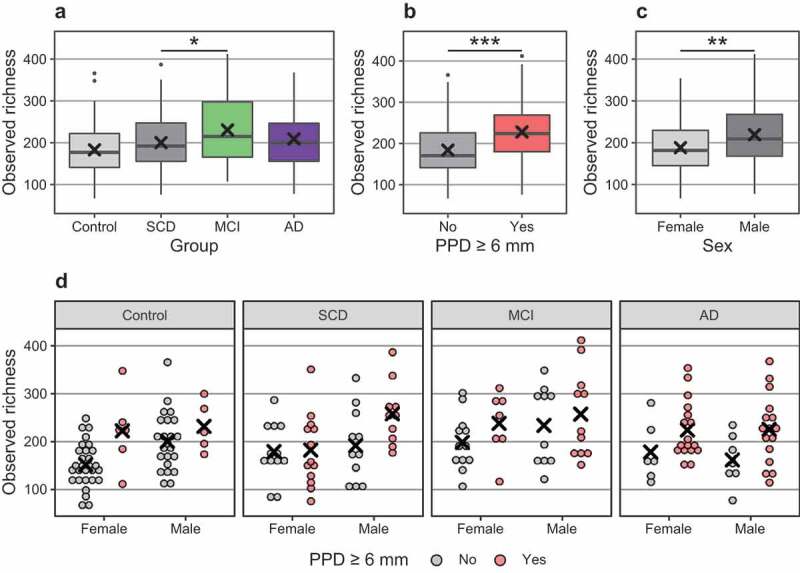
Differences in observed richness (number of detected bacterial OTUs) between groups. **Note**: x: mean. In boxplots, box hinges: 1st and 3rd quartiles, whiskers: box hinge to 1.5 * interquartile range. (a). Controls and diagnostic subgroups; (b). PPD ≥ 6 mm; (c). Sex; (d). Diagnostic subgroups, sex and PPD ≥ 6 mm

The richness and evenness of the microbial communities (alpha diversity) were estimated by using observed richness (number of OTUs) and Shannon index (Table 2). By using either index, diversity was higher in cases than in controls (p = 0.008 and p = 0.010) and differed across the four groups (p = 0.018 and p = 0.002). As seen in (Figure 2(a)), the subgingival microbiome was especially rich with a high diversity among participants with MCI. Among the possible confounders, alpha diversity (both indices) was higher in males than in females, and observed richness was higher in participants with PPD ≥ 6 mm (Table 2; Figure 2(b-c)). The association between alpha diversity and cognitive dysfunction was examined by using a linear regression model (Table S1). Male sex and PPD ≥ 6 mm were positive predictors of alpha diversity (Table S1; Figure 2) also in these analyses. However, even after adjusting for sex and presence of PPD ≥ 6 mm, MCI was significantly (p = 0.014) associated with alpha diversity (Table S1).

**Table 2.** P-values for differences in microbial alpha and beta diversity measures between variables of interest and potential confounders

	Alpha diversity: observed richness	Alpha diversity: Shannon index	Statistical test used for alpha diversity	Beta diversity: Bray-curtis dissimilarity,test: PERMANOVA
**Diagnosis**	**0.0175**	**0.0016**	Kruskal-Wallis	**0.0411**
**Case group vs control group**	**0.0081**	**0.0095**	Kruskal-Wallis	**0.0145**
**Age**	0.5754	0.7323	Pearson correlation	0.0644
**Sex**	**0.0049**	**0.0324**	Kruskal-Wallis	0.2649
**Education**	0.5723	0.1280	Kruskal-Wallis	**0.0430**
**Income**	0.7608	0.4051	Kruskal-Wallis	0.0827
**BMI**	0.2573	**0.0391**	Pearson correlation	**0.0088**
**Smoking**	0.2934	0.1079	Kruskal-Wallis	**0.0178**
**Few teeth**	0.4237	0.4869	Kruskal-Wallis	0.1544
**PPD 6 mm**	**0.0000**	0.7961	Kruskal-Wallis	**0.0001**

**Note**: Diagnosis: four-category variable based on AD, MCI, SCD and control group. Case vs control: two-category variable based on the combined case participant group (*i.e*. AD, MCI and SCD combined) and control group. Age: continuous variable. Gender: dichotomous variable. Education: 1–12 years of education or university education. Income: four-category variable based on gross annual income. BMI: continous variable. Smoking: three-category variable (current, previous or never). Few teeth: < 20 or ≥ 20 teeth. PPD 6 mm: ≥ 1 site with PPD of ≥ 6 mm; yes or no. Bold indicates statistical significance.

**Figure 3.**
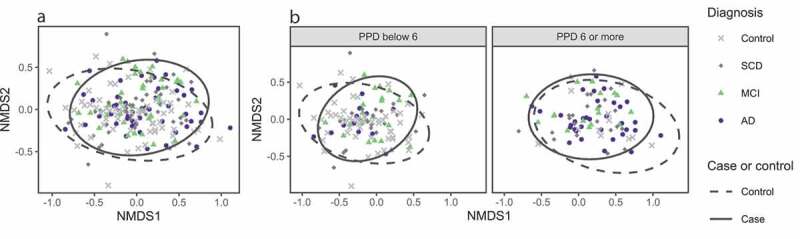
Non-metric multidimensional scaling plots based on Bray-Curtis dissimilarity. Each point corresponds to one participant; the closer the points, the more similar their samples. Ellipses represent 95% confidence intervals. (a). Colors and shapes for diagnostic case subgroups groups, ellipses for study group affiliation (all cases and controls). (b). Plot split by PPD ≥ 6 mm, with colors and shapes for diagnostic case subgroups and ellipses for study group affiliation (all cases and controls)

Differences in the microbial community composition (beta diversity) between the study groups were analysed using PERMANOVA. The differences were significant both between cases and controls (p = 0.014) and across the four groups (p = 0.041) (Table 2; Figure 3). Among the possible confounders, beta diversity differed most between the participants with or without PPD ≥ 6 mm (p < 0.001; Figure S1), but also between participants with different education levels, BMI or smoking habits. The association of beta diversity with cognitive dysfunction was further investigated with confounder-corrected models (Table S2). Also, in these analyses, BMI, smoking and PPD ≥ 6 were all associated with beta diversity (Table S2). However, after adjusting for possible confounders, the study group affiliation was associated with beta diversity (p = 0.030).

### Specific bacterial taxa

#### Cognitive dysfunction

The relative abundance of the detected subgingival bacteria was analysed next. The most common genera in the whole population were *Fusobacterium, Porphyromonas, Capnocytophaga, Treponema, Prevotella, Campylobacter* and *Streptococcus*, and their profiles did not differ between cases and controls (Table 3).

**Table 3.** Relative abundance (%, mean ± SD) for the ten most common bacterial genera in groups of participants

	Relative abundance (%, mean ± SD)				
	Controls	Cases	AD	MCI	SCD
***Fusobacterium***	17.97 ± 11.54	19.64 ± 11.77	17.62 ± 11.69	17.40 ± 9.02	23.61 ± 13.08
***Porphyromonas***	6.16 ± 16.20	5.58 ± 11.17	7.30 ± 13.12	5.37 ± 11.38	4.04 ± 8.59
***Capnocytophaga***	5.47 ± 5.14	6.10 ± 5.59	6.14 ± 5.89	6.27 ± 5.20	5.90 ± 5.72
***Treponema***	4.56 ± 4.91	5.38 ± 5.75	6.64 ± 7.16	5.55 ± 5.51	3.97 ± 3.85
***Prevotella***	3.91 ± 5.21	5.21 ± 4.30	5.21 ± 4.79	5.43 ± 3.17	5.02 ± 4.71
***Campylobacter***	4.25 ± 2.56	4.75 ± 2.57	4.48 ± 2.43	4.60 ± 2.12	5.14 ± 3.02
***Streptococcus***	6.29 ± 6.38	4.16 ± 4.68	4.08 ± 5.06	4.22 ± 3.74	4.19 ± 5.10
***Corynebacterium***	4.96 ± 6.39	4.46 ± 4.90	3.94 ± 5.08	4.84 ± 3.95	4.65 ± 5.48
***Rothia***	6.18 ± 9.40	3.95 ± 7.52	3.71 ± 8.55	4.38 ± 8.02	3.81 ± 5.95
***Actinomyces***	5.66 ± 7.29	3.30 ± 3.44	2.91 ± 3.15	3.00 ± 3.11	3.93 ± 3.95

The package DESeq2 was applied to further search for intergroup differences in OTUs, genera and families. After adjustment for age, BMI and PPD ≥ 6 mm, ten taxa differed significantly in abundance between case participants and controls (Table S3). Most notable among the OTUs, *Rothia aeria* (log2 fold change: −2.68, p < 0.001), *Corynebacterium durum* (log2 fc: −2.35, p < 0.001) and several members of *Actinomyces* genus (log2 fc: −1.56 − −1.04, p < 0.05) were more abundant in controls than in case participants. Only *Prevotella oulorum* (log2 fc: 1.80, p < 0.001) was more abundant in case participants than in controls. Similar differences were observed when the AD group was compared to controls. Moreover, two OTUs were specifically more abundant in the AD group than in controls: *Slackia exigua* (log2 fc: 2.80, p = 0.019), which was also associated with PPD ≥ 6 mm, and *Lachnospiraceae* [G-7] bacterium (log2 fc: 5.78, p < 0.001) (Table S3A).

The results at the family level were similar to the genus and OTU levels; for example, the family *Actinomyces* was less common in AD than in control participants (Table S3A).

#### Periodontal conditions

Subjects with PPD ≥ 6 mm had a higher abundance of 15 genera and a lower abundance of 13 genera (Figure S2, Table S3B). Among participants with PPD ≥ 6 mm, the highest log2-fold changes were identified for *Porphyromonas* (log2 fc: 2.80, p < 0.001) and *Peptostreptococcaceae* [XI] [G-5] (log2 fc: 2.52, p = 0.034). A total of 29 different OTUs were elevated in subjects with PPD ≥ 6 mm and the highest log2-fold changes were for *P. gingivalis* (log2 fc: 3.79, p < 0.001) and *Prevotella intermedia* (log2 fc: 2.55, p = 0.028). Several OTUs occurred less frequently in participants with PPD ≥ 6 mm than in those without, especially *Actinomyces massiliensis* (log2 fc: −2.12, p < 0.001), *Haemophilus parainfluenzae* (log2 fc: −2.08, p < 0.001), and *Streptococcus mutans* (log2 fc: −1.77, p = 0.021) (Table S3).

## Discussion

This study was undertaken in order to investigate the subgingival microbiota in persons with cognitive dysfunction (*i.e*. AD, MCI and/or SCD) in comparison with cognitively healthy controls. Overall, moderate intergroup differences emerged. Confounder-corrected models suggested that using either alpha or beta diversity measures, the cognitive dysfunction is a significant determinant of subgingival microbial composition and it is associated with higher microbial richness. In addition, the abundance of ten taxa differed significantly between case participants and controls.

However, the strongest determinant of the microbial diversity was periodontal disease, *i.e*. PPD ≥ 6 mm. This pathology was associated with a higher alpha diversity, observed richness and abundance of typical periodontitis-associated genera. These findings are in accordance with earlier studies [9]. The subgingival compartment is a highly specific niche in which the microbiota is to a large extent influenced by periodontal disease [33]. Prior studies have described the oral microbiota of the elderly, showing that age is associated with shifts in the microbiota [34]. In the present study, *Actinomyces* and *Rothia* were more common among controls with healthier periodontal status than in the diagnostic subgroup of AD with poorer periodontal status. The result is in accordance with earlier reports of an association between periodontal health and a greater abundance of *Actinomyces* and *Rothia* [9,35].

There is little published research on the subgingival microbiome in relation to cognitive function and dementia. One cross-sectional study using *16S rRNA* gene sequencing reported an association between impaired cognitive function and alterations in the subgingival microbiome [36]. Although the small sample size and study design precluded generalizable conclusions, the results suggested that there are consistent alterations in the microbiota among participants with dementia compared with those without dementia. An interesting finding in the present study is the increased abundance of *Lachnospiraceae*. This taxon has also been shown to be increased in the gut microflora of AD patients [37] and to associate with several inflammatory conditions, such as metabolic syndrome, obesity, diabetes, liver diseases, inflammatory bowel disease and chronic kidney disease [38].

Studies using other approaches to detect microbial signatures have shown that increased systemic levels of antibodies against specific periodontal pathobionts are associated with impaired cognitive function. In a large study, individuals with the highest serum *P. gingivalis* antibody levels were at increased risk of impaired cognitive function [14]. A study analysing *postmortem* brain tissue reported that *P. gingivalis* lipopolysaccharides (LPS) could reach the brain [39]. Also, spirochetes, especially *Treponema* species, have been implicated in AD pathogenesis [40]. In our previous study, we showed that signs of periodontal disease were more common in the case groups, especially the AD group [16]. In the present study, PPD ≥ 6 mm was associated with several OTUs representing established periodontal pathogens, such as *P. gingivalis, Tannerella forsythia, Treponema denticola* and *P. intermedia*. This provides support that these bacteria are linked with AD. Future studies will show whether oral dysbiosis, dysfunctional host response or both, contribute to AD risk.

In interpreting the results of this study, several limitations should be considered. As periodontal disease was more common in case participants than in controls, an independent association between cognitive impairment and microbial signatures cannot be determined. In order to distinguish microbial signatures more readily, future microbial surveys exploring the subgingival microbiota in AD should aim to recruit participants in whom the periodontitis burden is more evenly distributed. Furthermore, the participants were recruited irrespective of smoking status or previous antibiotic or periodontal treatment, all of which have profound impact on the subgingival microbiota [41]. Another limitation is the sampling method. Subgingival samples were obtained using curette sampling from the deepest periodontal pockets. This does not result in a quantitative sample representing all subgingival microbiota. However, different methods for subgingival sampling seem to show good agreement [42].

## Conclusion

The results provide evidence for differences in the subgingival microbiota between individuals with cognitive dysfunction and cognitively healthy individuals. The alterations are mainly attributable to the higher prevalence of periodontal disease in the groups with different degrees of cognitive dysfunction. Altogether, these findings are of relevance to our understanding of the association between periodontal disease and cognitive dysfunction.

## Supplementary Material

Supplemental Material
