# Supplementary material for: Subgingival microbiota in a population with and without cognitive dysfunction
Source: J Oral Microbiol. 2021 Jan 15;13(1):1854552. doi: 10.1080/20002297.2020.1854552 (PMC7833025; doi:10.1080/20002297.2020.1854552)
Supplement: Supplemental Material [file ZJOM_A_1854552_SM2742.zip › Supplementary/Supplementary Legend.docx]

# **Supplementary material**

## **Figure S1.** Non-metric multidimensional scaling plots based on Bray-Curtis dissimilarity. Ellipses represent 95% confidence intervals. **A**. Samples (each point corresponds to one participant), split by study group affiliation, colours and shapes according to PPD ≥ 6 mm. **B**. Operational Taxonomic Units (each point corresponds to one taxon), showing OTUs belonging to the 10 most abundant genera, coloured by genus. Plots **A** and **B** depict the same analysis, *e.g.* samples with a low NMDS1 value in **A** have high amounts of the bacteria with a low NMDS1 value in **B**.

## **Figure S2.** Boxplots of bacterial genera with abundance differences for the PPD ≥ 6 mm variable for dental health. Box hinges: 1st and 3rd quartiles, whiskers: box hinge to 1.5 * interquartile range.

## **Table S1.** Results for linear regression of observed richness and variables of interest.

## **Table S2.** Results for PERMANOVA with multiple variables. **A**. Full model; **B**. Trimmed model with significant variables from the full model.

## **Table S3.** Full results of differential abundance comparisons with DESeq2. **A**. All cases/control and diagnostic subgroups; **B**. Periodontal health variables.
